# Imbalance learning for the prediction of N^6^-Methylation sites in mRNAs

**DOI:** 10.1186/s12864-018-4928-y

**Published:** 2018-08-01

**Authors:** Zhixun Zhao, Hui Peng, Chaowang Lan, Yi Zheng, Liang Fang, Jinyan Li

**Affiliations:** 1 Advanced Analytics Institute, Faculty of Engineering and Information Technology, University of Technology Sydney, PO Box 123, Broadway, Sydney, NSW 2007 Australia; 2 School of Computer, National University of Defense Technology, Changsha, 410073 China

**Keywords:** N^6^-methyladenosine, Site Prediction, Imbalance Learning

## Abstract

**Background:**

N^6^-methyladenosine (m^6^A) is an important epigenetic modification which plays various roles in mRNA metabolism and embryogenesis directly related to human diseases. To identify m^6^A in a large scale, machine learning methods have been developed to make predictions on m^6^A sites. However, there are two main drawbacks of these methods. The first is the inadequate learning of the imbalanced m^6^A samples which are much less than the non-m^6^A samples, by their balanced learning approaches. Second, the features used by these methods are not outstanding to represent m^6^A sequence characteristics.

**Results:**

We propose to use cost-sensitive learning ideas to resolve the imbalance data issues in the human mRNA m^6^A prediction problem. This cost-sensitive approach applies to the entire imbalanced dataset, without random equal-size selection of negative samples, for an adequate learning. Along with site location and entropy features, top-ranked positions with the highest single nucleotide polymorphism specificity in the window sequences are taken as new features in our imbalance learning. On an independent dataset, our overall prediction performance is much superior to the existing predictors. Our method shows stronger robustness against the imbalance changes in the tests on 9 datasets whose imbalance ratios range from 1:1 to 9:1. Our method also outperforms the existing predictors on 1226 individual transcripts. It is found that the new types of features are indeed of high significance in the m^6^A prediction. The case studies on gene c-Jun and CBFB demonstrate the detailed prediction capacity to improve the prediction performance.

**Conclusion:**

The proposed cost-sensitive model and the new features are useful in human mRNA m^6^A prediction. Our method achieves better correctness and robustness than the existing predictors in independent test and case studies. The results suggest that imbalance learning is promising to improve the performance of m^6^A prediction.

**Electronic supplementary material:**

The online version of this article (10.1186/s12864-018-4928-y) contains supplementary material, which is available to authorized users.

## Background

Among more than 140 kinds of post-transcription modifications (PTMs) [1, 2], N^6^-methylation (m^6^A)—the methylation at 6th nitrogen of adenosine, is one of the most abundant modifications [3, 4]. This methylation has been widely found in species such as Arabidopsis thaliana, Saccharomyces cerevisiae, bacteria, virus, human, and mouse [5–8]. More exactly, these methylation events have occurred in the mRNAs at the 3’ untranslated regions (UTRs) close to the stop codon, following a conserved sequence motif DRACH, [G/A/C] [G/A] A^*^ C [U/A/C], (where A^*^ stands for the m^6^A site) [9, 10]. The dynamic m^6^A methylation involves many proteins such as METTL3, METTL14, WTAP, ALKBH5 and YTHDF2 [3, 11–13]. With intensive investigation on this dynamic and reversible methylation in mRNAs recently, the functions of m^6^A in biological processes have been significantly redefined. It is reported that m^6^A disruption can effect translation efficiency [14], cell viability [15] and cell development [11]. The level changes of m^6^A in mRNA can lead to abnormality of RNA export, protein translation or RNA editing, causing cancer, obesity, and other human diseases [16–19]. For example, strong relationships have been observed between m^6^A and HIV-1 [20, 21], Zika virus infection [22] and breast cancer stem cell phenotype [23]. The identification of m^6^A sites is crucial for understanding the disease mechanisms and identifying novel medicine targets.

Experimental approaches including two-dimensional thin layer chromatography [24], high performance liquid chromatography [25], and high-throughput methods (e.g., m^6^A-seq [9] and MeRIP-Seq [10]) have been applied to identify m^6^A sites in mRNAs. However, they can only detect m^6^A-containing transcript fragments instead of identifying the exact methylated adenines [26]. Based on the single-nucleotide resolution m^6^A maps in mRNAs, researchers have explored computational methods with sequence features and machine learning algorithms to make m^6^A sites prediction. For instance, iRNA-Methyl [27], m^6^Apred [6] and RAM-ESVM [28] are predictors aiming at yeast m^6^A site prediction [29]; methods SRAMP [30], Methy-RNA [31] and RAM-NPPS [32] are built on human and mouse m^6^A maps [33, 34]. There are also some predictors developed for Arabidopsis thaliana [35–37].

One critical issue of this challenging prediction problem is that non-m^6^A sites are much more than m^6^A sites in the training data. The existing computational methods have overlooked this imbalance issue. In fact, they trained the model with balanced datasets containing roughly equal sizes of m^6^A samples and randomly selected non-m^6^A samples. Such sampling of non-m^6^A samples may lead to inadequate learning and the prediction models would change when the selected non-m^6^A samples are different.

Here we use a cost-sensitive XGboost classifier to address the imbalance issue. Similarly as previous works, m^6^A samples and non-m^6^A samples are labeled as positive and negative respectively. The classifier is then trained with all the samples without selecting a subset of negative samples and prevents over-fitting by defining different costs for the misclassified positive and negative samples. In the learning stage, the model minimizes the cost function and improves the precision of classifying positive samples. Besides, ROC rather than accuracy is set as the training cost function. Owing to training on the whole dataset without sampling noise, our method which is called HMpre, exhibits higher performance and better robustness.

Another issue of computational m^6^A prediction is the lack of valid features. The state-of-the-art features are usually derived from window sequences with m^6^A at the centre position. These features include binary encoding sequence features [30, 35], k-mers [35], physical-chemical properties [38, 39], position-specific nucleotide propensities [40], pseudo nucleotide compositions [28, 41, 42], nucleotide pair spectrums [30] and multi-internal nucleotide pair positions [32].

To improve the effectiveness of feature space, we present three types of novel m^6^A features. First, we extract novel features to capture specific single nucleotide polymorphism (SNP) variants in the window sequences through the MRMR method and Fisher’s exact test [43]. These features are relevant because single nucleotide variants can effect m^6^A dynamics [44]. Moreover, m^6^A occurs richly in some particular regions of transcripts, thus we calculate the absolute and relative locations of m^6^A sites as new features. To further exploit the distribution properties of nucleotides, entropy information is also considered as new features. Together with these newly proposed features, conventional features including 4-bit binary, overlapping chemical property with density and k-mers are integrated into our feature space to describe comprehensive characteristics of methylation.

In the performance evaluation of our method HMpre, we first report specific SNP positions as new features. Then we report a detailed comparison result with three existing balance learning predictors on an independent test dataset. HMpre achieves a much better performance of precision 0.3035, F1 0.3961 and MCC 0.3329. Since the ratio of positive sites over negative sites in a test mRNA is unknown, HMpre and existing predictors are also evaluated on 9 datasets containing different ratios of positive sites over negative sites. Results show that HMpre works better and has stronger robustness on the ratio change. In practical use, the inputs to a predictor are always individual transcripts, therefore the four methods are then applied to make predictions on single transcripts. Again, HMpre achieves the best overall performance. Furthermore, we evaluate the features effectiveness with 10-fold cross validation and feature importance scores from XGBoost classifier. The new features are all meaningful and the proposed feature space improves performances notably. In the case studies, the transcript of c-Jun gene is taken as an example to demonstrate the prediction details. Then we evaluate our method on the transcript of CBFB gene relating to HIV-1 infection and our method also achieves better results than the other predictors.

## Methods

### Datasets

Currently validated human mRNA m^6^A sites were all obtained by Ke and Linda from single nucleotide resolution maps [33, 34]. To guarantee the reliability of negative samples, non-m^6^A sites conforming to the conserved motif DRACH were all produced from these validated transcripts. Based on these datasets, Zhou has built a human mature mRNA m^6^A dataset which is the largest human m^6^A dataset so far. The dataset used in our experiments is downloaded from Zhou’s work [30]. After removing redundant and unaligned samples, we get 7506 human mature transcripts in total. We reserved 6280 transcripts for training and 1226 transcripts for independent testing. For each transcript, the number of non-m^6^A conforming to the DRACH motif is much larger than m^6^A sites. The training dataset contains 26512 positive samples and 271214 negative samples, while the independent test dataset contains 5644 positive samples and 54744 negative samples. Each sample contains the transcript id, the location of target adenine and the flanking window sequence. All samples used in our dataset are listed in Additional file 1.

### Feature space construction

**Fig. 1:**
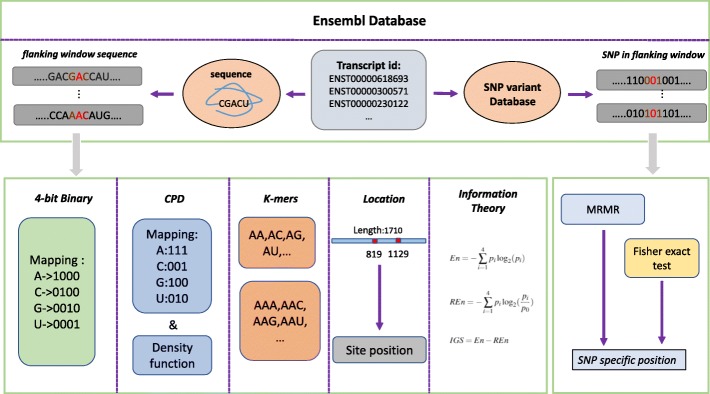
Feature Space Construction

Computational prediction methods usually build features from a flanking window sequence with m^6^A at the centre position. The size of the flanking window varies from 20 to 50 nts in previous works and we choose the size of 25-nt which is similar to other human predictors. Thus the features are extracted from the 51-nt long sequence. Based on the sequence characteristics of m^6^A site, we introduce three types of new features: site location related features, features related to entropy information and SNP features. Three types of conventional features are also used. There are totally 509 dimensions in our feature space. The transcript sequences, length information (including coding region and UTRs) and SNP variants are obtained from the Ensembl online human gene database (GRCh38.p10). A diagram of the feature space construction is presented in Fig. 1.

#### Three new types of sequence features

*Site Location Related Features* In mature transcripts, m^6^A sites are rich in some special regions, such as the 3’ UTRs near the stop codon [10]. However, non-m^6^A sites conforming to the DRACH motif are randomly distributed over the entire transcript. Thus the location of target adenine site in the transcript can be taken as a new feature. Specifically, site location refers to the distance between the target site and the transcript start site. Beside, the relative location of the target site in the whole transcript is also taken as a new feature, which is the ratio of the site location over the transcript length.

*Features Related to Entropy Information* Because of motif conservation for regulating protein binding sites, the nucleotides around m^6^A sites have some unique distributions. Shannon information theory can be used to evaluate these nucleotide distributions in the transcript fragment sequences. We calculate Shannon entropy (En), relative entropy (REn) and information gain score (IGS) of all samples as a new type of feature. The scores of these features are calculated as:

1 $$ En(s)=-\sum\limits_{i \in \{A, G, U, C\}}p_{i}^{s}\log_{2}(p_{i}^{s})  $$

2 $$ REn(s)=-\sum\limits_{i\in\{A, G, U, C\}}p_{i}^{s}\log_{2}(\frac{p_{i}^{s}}{p_{0}})  $$

3 $$ IGS(s)=En(s)-REn(s)  $$

where $p_{i}^{s}$ is the frequency of A, G, U, C in sequence *s*, and *p*_0_ is the uniform distribution of each nucleotide occurrence, namely *p*_0_=1/4. The frequency of each nucleotide is then combined with the entropy features as a 7-dimension feature vector.

*SNP Features* Singe nucleotide polymorphism is a kind of variant at specific sites in genome. For SNP sites, several possible nucleotide variations are alleles for this position. As a synonymous single nucleotide variant, SNP changes the sequence of mRNA but does not alter the amino acid sequence of protein [45]. In addition, m^6^A is regulated by some proteins which also have fixed RNA binding sites, which means the flanking window sequence around m^6^A site has specific base groups patterns. The SNP variant of mRNA sequence may disrupt the DRACH motif or protein binding regions, leading to failures of m^6^A dynamic regulations [44]. Hence, we attempted to find positions with unique SNP states. From the Ensembl database, we map SNP variants in the transcript and convert sample sequence into a 51-bit 0/1 vector (i.e., 0 denotes a non-SNP variant position; 1 donates an SNP variant position). As there are various methods to select effective features [46, 47], in this paper Max-Relevance Min-Redundancy (MRMR) algorithm [43] and Fisher’s exact test are adopted to recognize special SNP positions.

MRMR selects positions with a maximal statistical criterion based on mutual information. MRMR tries to find a position subset, which have maximum relevance (dependency) with class and minimum internal redundancy. MRMR adds positions into the subset one by one and the order is determined by relevance to the target class and the redundancy with the other positions. Fisher’s exact test is a statistical significance test. For an individual position, it investigates the SNP variant distribution difference between the positive and negative samples and derives a p-value to assess the difference. A low p-value means the SNP variant at this position has great difference between the negative and positive samples. Finally, we can rank positions with Fisher’s exact test p-value and the MRMR selection order. By calculating the average ranking of MRMR and Fisher’s exact test, positions with a significant SNP specificity can be identified. The SNP variant states of such specific positions are considered as SNP features. The detailed SNP specificity identification algorithm is presented in supplementary Algorithm S1.

#### Conventional sequence features

*4-bit Binary Features* Binary encoding is a common feature extraction method to characterize RNA sequences. As mRNA sequence contains four nucleotides A, C, G and U, this encoding method can map every single nucleotide into a 4-bit binary code. The mapping rules are: ’A’- (1,0,0,0), ’C’- (0,1,0,0), ’G’- (0,0,1,0), ’U’- (0,0,0,1). By this way, a 51-nt sequence can be transformed into a 204-dimension feature vector.

*Chemical Property with Density (CPD)* Based on differences in chemical property, four kinds of nucleotides can be categorized into different groups [48]. In terms of ring numbers in a single base group, C and U have only one ring while A and G have two. Besides, C and G have strong hydrogen bonds when forming secondary structures, whereas hydrogen bonds in A and U are both weak. When considering chemical functionality, amino group contains A and C while keto group includes G and U. Thus, we can divide the nucleotides by different chemical properties and use overlapping encoding rules: ’A’- (1,1,1), ’C’- (0,0,1), ’G’- (1,0,0), ’U’- (0,1,0). In literature work, the density of nucleotide is always used with chemical property features, which calculates the frequency of a nucleotide occurring before current position. Density feature *d_i* is defined as:

4 $$ d_{i} =\frac{1}{\left | S_{i} \right |} \sum\limits_{j=1}^{i}f(s_{j}), f(s_{j})=\left\{\begin{array}{ll} 1 & s_{j} = s_{i}\\ 0 & s_{j} \neq s_{i} \end{array}\right.  $$

*K-mer Features* In mRNA sequence, adjacent nucleotide pairs have influence on mRNA structures and functions. K-mer is the frequency of k-nt adjacent nucleotides. As a global feature, k-mer has been proved to be effective in many sequence based site predictions. The length of k-mer feature is *4*^*k*^ bits. In this paper, we adopt 2-mer and 3-mer. Each sample has a 80-dimension k-mer feature vector.

### Imbalance learning

Imbalance learning has been explored for protein binding site prediction [49–51] and protein-protein interaction sites identification [52, 53]. However, the imbalance learning for m^6^A prediction has not been explored. An intuitive way to address this problem is to integrate sampling and ensemble techniques, which trains basic classifiers with different sampling data and combines the results in an ensemble way to reduce the random sampling bias. But it requires effective sampling techniques to select meaningful negative subsets and there are some researches focus on dynamic and cluster ways [54]. Another viable strategy is to introduce cost-sensitive learning models, like weighted support vector machine and cost-sensitive decision trees, using different matrices to describe the costs for classifying sample into wrong class [55].

Here we use a cost-sensitive XGBoost classifier as learning model. XGBoost (eXtreme Gradient Boosting) is a tree boosting algorithm developed by Chen [56]. It is an advanced implementation of gradient boosting algorithm, which has been widely applied for classification problems. XGBoost has some advantages over other cost-sensitive classifiers. Firstly, the regularization can effectively prevent training model from over-fitting. Secondly, embedded parallel processing allows a faster learning speed. Thirdly XGBoost is of high flexibility and allows users to define custom optimization objectives and evaluation criteria. Moreover, XGBoost classifier can learn from imbalance training data by setting class weight and taking ROC as evaluation criteria. Here we implement the model with a python package named xgboost (vision 0.6a2). The parameters can be optimized by 10-fold cross validation in the learning stage. The parameters in our model are: ’lambda’: 700, ’max-depth’: 6, ’eta’: 0.1, ’silent’: 1, ’objective’: ’binary:logistic’, ’booster’: ’gbtree’,’scale-pos-weight’: 6, ’eval-metric’: ’auc’ and training boost round is 400, while other parameters are all default values.

In this paper, our method is compared with three recently published human m^6^A prediction methods. These three literature methods are: SRAMP [30], Methy-RNA [31] and RAM-NPPS [32]. They all have open access web predictors and SRAMP also provides a tool package for local implementation. The prediction results of Methy-RNA and RAM-NPPS are obtained from the web predictors, while the results of SRAMP are derived from tool package in mature mode.

### Performance evaluation metrics

The proposed prediction method is evaluated by 10-fold cross validations and independent test dataset with four frequently used metrics: precision, recall, F1-score and Matthews correlation coefficient(MCC). As RAM-NPPS and Methy-RNA cannot return prediction probabilities, we do not use AUROC or AUPRC as evaluation metrics.

Precision and recall reflect the tendencies of classifier prediction. Recall (also called sensitivity in binary classification) illustrates how many positive samples are rightly classed, and precision shows the ratio of true positive sample ratio in all predicted positive-label samples. There is always a trade-off between precision and recall, so we introduce F1 and MCC to evaluate the overall performance of a predictor. F1-score combining precision and recall together can assess the performance on both balanced and unbalanced test datasets. MCC is also a frequently used metric in classifier evaluation, which return a value between -1 to 1: 1 standing for perfect prediction and -1 for reversed prediction.

## Results

We report the specificity results of SNP identification as new features. In the performance comparison and evaluation, we tested our HMpre method and other existing predictors on the independent test dataset. To demonstrate the robustness of our method to deal with the unknown percentages of positive samples in real transcripts, we compared our method with three existing human m^6^A predictors on datasets of different positive-and-negative sample ratios. To evaluate the performance for the practical use, we tested all the predictors on single transcripts. Lastly, we report the feature effectiveness results of HMpre and XGBoost classifier feature importance scores.

### Specific SNP identification as new features

**Fig. 2:**
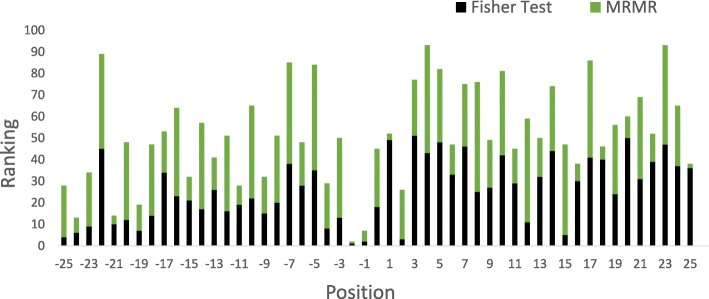
SNP Specificity Ranking. The black blocks stand for the Fisher’s exact test rankings and the green blocks stand for the MRMR rankings. X-axis is the window sequence sites from -25 to 25. Y-axis is the total ranking of each position. A low ranking means a high SNP specificity at this position

To identify positions with specific SNP variant states as new feature, MRMR and Fisher’s exact test are applied to analyze sequence SNP variant states in the training dataset. As presented in Fig. 2, MRMR and Fisher’s exact test give rankings to all the positions numbered from -25 to 25 in the window sequence.

In the process of selecting a position subset, MRMR defines mutual information to evaluate the subset for the inner redundancy and relevance with the target class, then it gives out the order of position selection and we take the order as position importance ranking. The top 12 positions are -2, 25, 1, -21, -1, 18, -24, 16, -11, 20, -15 and -19. Fisher’s exact test can statistically recognize the SNP variant distribution difference for these individual positions between the positive and negative samples, as described by a p-value. With Fisher’s exact test p-values (details in Table S1 of Additional file 2), we can also rank all these positions. The top 12 positions are -2, -1, 2, -25, 15, -24, -19, -4, -23, -21, 12 and -20. Finally we choose the top 12 positions with the highest average ranking as SNP features. These highly ranked positions are illustrated in Table 1. These positions have relatively higher ranking both in MRMR and Fisher’s exact test. Detailed results are listed in Table S2 of Additional file 2.

**Table 1:** Ranking details of Top 12 specific SNP positions (FET: Fisher’s exact test)

No.	Position	FET ranking	MRMR ranking	Average	Ranking
1	-2	1	1	1	1
2	-1	2	5	3.5	2
3	-24	6	7	6.5	3
4	-21	10	4	7	4
5	-19	7	12	9.5	5
6	2	3	23	13	6
7	-25	4	24	14	7
8	-11	19	9	14	7
9	-4	8	21	14.5	8
10	-15	21	11	16	9
11	-9	15	17	16	9
12	-23	9	25	17	10

### Performance on the independent dataset

Our proposed HMpre is compared with three existing prediction methods on the independent test dataset. The prediction results are reported in Table 2. HMpre achieves the best performance under all metrics except recall; RAM-NPPS has a better recall of 0.6339 than HMpre. The precision of HMpre is 0.3035, 0.04 higher than SRAMP which is the best in the existing predictors. Overall, HMpre achieves F1 score of 0.3961, higher than the best F1 value of the other three predictors (0.3408 by RAM-NPPS). In terms of MCC, Methy-RNA has a value of -0.1619 and SRAMP is 0.2653, about 0.08 higher than RAM-NPPS, but still lower than HMpre’s 0.3329.

**Table 2:** Performance on the Independent Test Dataset (Methy: Methy-RNA; NPPS: RAM-NPPS)

Methods	Precision	Recall	F1	MCC
Methy	0.065	0.5184	0.1163	-0.1619
NPPS	0.1656	**0.6339**	0.2626	0.1833
SRAMP	0.2638	0.4812	0.3408	0.2653
HMpre	**0.3035**	0.5698	**0.3961**	**0.3329**

These data in boldface just means the largest values in each metrics

### Robust performance when tested on datasets with different imbalance ratios

**Fig. 3:**
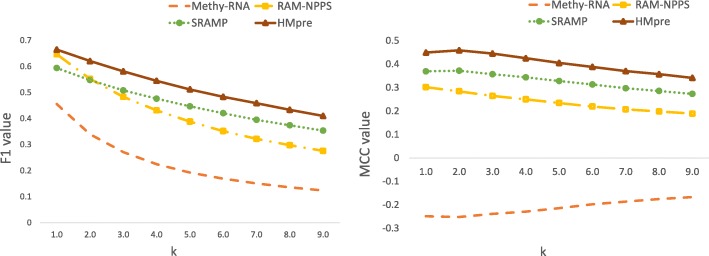
Performance on Datasets of Different Imbalance Levels. The F1 and MCC values of four predictors are represented. X-axis k is the ratio of the negative samples to positive samples (imbalance level) in a test dataset; Y-axis is metric value

In normal situations, the numbers of m^6^A and non-m^6^A sites are unknown before prediction. Therefore, a practical m^6^A predictor should have a strong robustness against the imbalance level change. To appraise the robustness of HMpre and other predictors, we test them on nine datasets whose negative samples to positive samples ratios range from 1:1 to 9:1. Here we adopt the overall metrics F1 and MCC as evaluation criterions. The results are reported in Fig. 3. The F1 and MCC values of all the methods have a trend of decreasing when the imbalance level increases. The F1 scores of RAM-NPPS and Methy-RNA decrease more rapidly than HMpre and SRAMP. For the MCC values, HMpre also has a relatively slow changing rate while the other methods are comparable. Moreover, HMpre has a better performance on all of these datasets under F1 and MCC, proving that HMpre has a stronger robustness.

### Average performance on 1226 individual transcripts

Since the testing objects are always single transcripts in real cases, the four predictors are evaluated on individual transcripts. There are 1226 transcripts in the independent dataset for the four methods to make predictions. The imbalance levels of the 1226 transcripts are different, and we calculate the average metric values of all the transcripts as the final results for each method. The results are reported in Table 3. Although RAM-NPPS has the highest recall of 0.6582, HMpre achieves the best performance under the remaining four metrics (precision 0.2972, recall 0.6062, F1 0.3658 and MCC 0.3239). Especially, the overall metrics F1 and MCC of HMpre are about 0.07 and 0.08 higher than SRAMP, the best existing predictor.

**Table 3:** Performance on Individual 1226 Transcripts (Methy: Methy-RNA; NPPS: RAM-NPPS)

Methods	Precision	Recall	F1	MCC
Methy	0.0723	0.5075	0.1174	-0.1614
NPPS	0.1770	**0.6582**	0.2529	0.1907
SRAMP	0.2484	0.4759	0.2928	0.2387
HMpre	**0.2972**	0.6062	**0.3658**	**0.3239**

These data in boldface just means the largest values in each metrics

### Feature importance analysis

Three types of new features are extracted to add to the existing feature space to improve the prediction performance. 10-fold cross validations with different feature spaces are used to verify whether the new feature space actually improves the prediction performance. The performance of the three types of traditional features and their merged features are compared with the proposed feature space in Table 4. The three types of traditional features (four-bits binary coding, chemistry property with density and k-mers) achieve distinct performance and the 4-bit binary features are better than the other two types of features. By joining the three types of conventional features together, all metrics increase comparing with individual features. The proposed feature space, combining conventional and new features together, exhibits the best performance under all metrics.

**Table 4:** Different Feature Space Performance in Cross Validation (CPD: Chemical Property with Density; Joint: joint of conventional features)

Feature	Precision	Recall	F1	MCC
K-mers	0.1392	0.3426	0.2461	0.1572
CPD	0.2460	0.4816	0.3256	0.2532
Binary	0.25	0.4906	0.3312	0.2601
Joint	0.2519	0.5035	0.3358	0.2661
Proposed	**0.2669**	**0.5248**	**0.3538**	**0.2877**

These data in boldface just means the largest values in each metrics

We also attempted to understand more about the role of each feature in prediction. XGBoost can make an inner analysis of feature importance during learning process and output scores for all the features. The importance scores can reveal how meaningful the features are when building model and tell which features plays leading roles in the feature space.

**Fig. 4:**
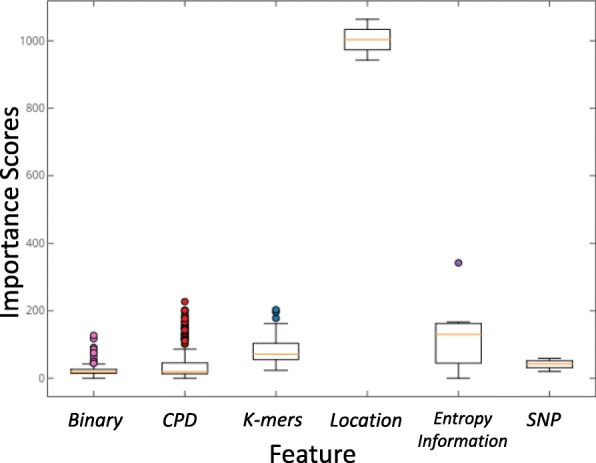
Boxplot of Feature Importance Scores

The feature importance scores boxplot is presented in Fig. 4. There are 509 features and their distribution is presented in Table S3 of Additional file 2. The importance scores have a wide range from 0 to 1064. The features with a 0 score are from 4-bit binary and CPD features, corresponding to the motif adjacent sites which are ’GAC’ or ’AAC’ in all the samples. The dimension with the highest score 1064 (f501) refers to the site distance from transcript start site, followed by features of relative location in transcript (f500, scored 943) and sequence entropy (f506, scored 342). Besides, density features in CPD features has relatively high importance scores. Detailed importance scores is shown in Figure S1. For the average score, binary and CPD are much lower than other features while site location and entropy information are obviously higher. K-mers and SNP have comparable average scores. From the results, the three types of new features are indeed significant in the feature space.

## Case studies

In this section, we report two detailed case studies to understand the difference of the four predictors and evaluate their capacity in practical use. First, we describe the prediction results for the c-Jun transcript from the test dataset. The second case study is about the m^6^A sites in the mRNAs of CBFB gene which can modulate HIV-1 replication and infection [20].

### m^6^A prediction for c-Jun transcript

**Fig. 5:**
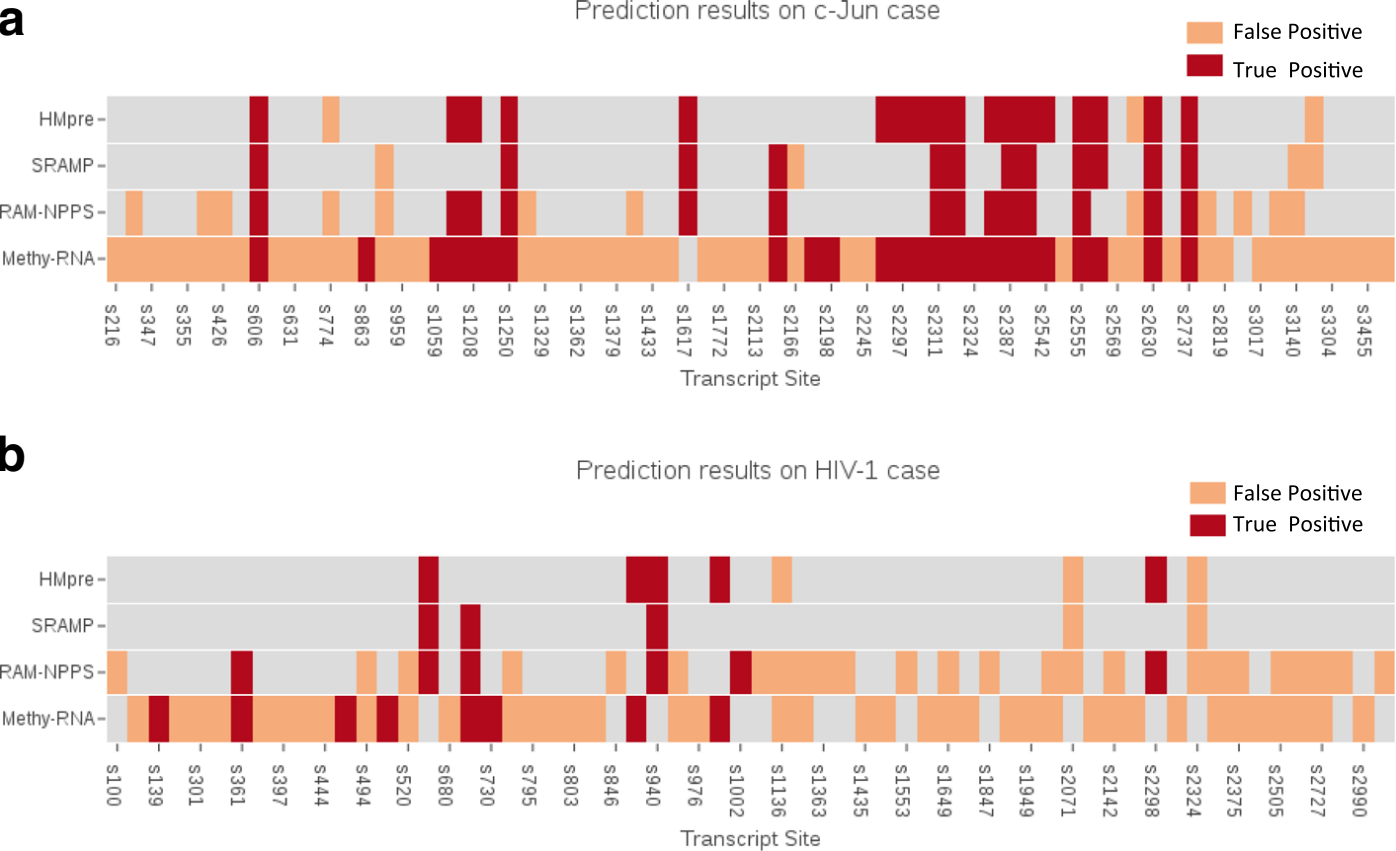
Predicted m^6^A sites in the case studies. The x axis stands for the potential m^6^A sites confirming to the sequence motif DRACH and the y axis indicates the four predictors. All colored blocks are the predicted m^6^A sites. Red blocks represent true positive sites and yellow blocks are false positive ones. (**a**) the prediction results for the c-Jun case and (**b**) the predictions for the HIV-1 case

Transcript ENST00000371222 of c-Jun gene contains 25 verified m^6^A sites and 47 non-m^6^A sites conforming to the DRACH motif. HMpre made a prediction of 21 m^6^A sites: 18 of them are true positives while 3 are false positives. SRAMP made 12 true positive m^6^A sites and 3 false positives. RAM-NPPS made 14 true positives and 12 false positives. Methy-RNA made the most 31 false positive predictions and identified only 19 true m^6^A sites. Thus, Methy-RNA achieved the highest true positive rate, but it made the most number of false positive predictions. See Fig. 5. Despite SRAMP achieved a good precision of predicted m^6^A sites, a large number of true m^6^A sites were wrongly classified. RAM-NPPS has more false positives and less true positive predictions than SRAMP and HMpre.

Table 5 shows the detailed prediction performance. Overall, the precision, F1 and MCC of our HMpre method are much higher than the other prediction methods. Although Methy-RNA has a high recall 0.96, it has the lowest precision, F1 and MCC. The performance of SRAMP is better than RAM-NPPS, but the recall of SRAMP is the lowest 0.48, suggesting a lot of positive samples are predicted to be negative.

**Table 5:** Results for the c-Jun gene case study (Methy: Methy-RNA; NPPS: RAM-NPPS)

Case	Methods	Precision	Recall	F1	MCC
c-JUN	Methy	0.3428	**0.96**	0.5052	-0.0542
	NPPS	0.5384	0.56	0.549	0.3019
	SRAMP	0.75	0.48	0.5853	0.4522
	HMpre	**0.8571**	0.72	**0.7826**	**0.6872**
HIV-1	Methy	0.1702	**0.6666**	0.2711	-0.1045
	NPPS	0.1935	0.5	0.279	0
	SRAMP	0.6	0.25	0.3529	0.2727
	HMpre	**0.6256**	0.4166	**0.5**	**0.4203**

These data in boldface just means the largest values in each metrics

### m^6^A site prediction for a transcript related to HIV-1 infection

The longest transcript ENST00000290858 of CBFB gene from the Ensembl database was chosen for this case study. There are 62 adenines (A) conforming to the motif in this transcript. The experimentally validated m^6^A sites of CBFB gene are acquired from RMBase, a online m^6^A database [57]. Based on these data, we constructed a test dataset of 12 positive samples and 50 negative samples.

The predicted m^6^A sites are presented in Fig. 5. HMpre made 5 true positive and 3 false positive predictions. SRAMP made 3 true positive and 2 false positive predictions. RAM-NPPS and Methy-RNA made more false positives than true positives: RAM-NPPS had 6 true positives and 16 false positives, and Methy-RNA had 51 false positives and 8 true positives. The predicted m^6^A sites by SRAMP are mainly correct, but it missed a lot of true m^6^A sites.

The detailed results are reported in Table 5. Methy-RNA achieves the best recall 0.6666 but the worst precision 0.1702. SRAMP has a high precision 0.7692 but the lowest recall 0.25. Our HMpre method has the best precision 0.56256 and achieves the best performance on the overall metrics F1 0.5 and MCC 0.4203.

## Discussion

In this paper, we adopted a XGBoost classifier as the prediction model. On one hand, this classifier can learn from imbalance data which is similar to data in practical prediction situations and inner regularization rules can prevent model from over-fitting; on the other hand, when the scale of training data is quiet large, it would cost classifiers like SVM and Random forest much longer time than our method in training stage.

The efficiency of features is crucial to the performance of predictors. Here, we presented m6A sites with meaningful biological features instead of just using flank window sequence features. In this work, the size of flanking window are fixed to 51-nts which is the same with existing methods. The influence of sequence size on feature efficiency will be studied in next stage of research. In addition, some m6A biological characteristics found recently can be taken as new features in the prediction and we will try them in the future.

## Conclusion

To address the problem of class imbalance in the training data for human mRNA m^6^A prediction, we have proposed a novel computational method called HMpre. The key idea is a cost-sensitive learning model. Three types of new features are also introduced to learn more from the imbalanced training data for the further improvement of the prediction performance. Along with other three existing methods, HMpre was tested on an independent dataset. The results show that our method has better correctness and robustness. The feature importance analysis demonstrates that the new features are exactly meaningful in the prediction. In the detailed cases studies, our method also outperforms over the existing predictors. Class imbalance is a long-neglected but important issue in the m^6^A prediction problem. Imbalance learning provides a promising way to resolve this issue.

## Additional files

Additional file 1 Data set of human mature mRNA N^6^-Methylation. Training and testing data used in this paper is accessible in this file. For each sample, the transcript id, site position, transcript length and flanking sequence with a size of 26 nts are given. (XLSX 15155 kb)

Additional file 2 Supplementary Tables, Algorithm and Figure. Table S1: The result of Fisher’s exact test on training data. The SNP variant states of positive and negative samples are counted respectively at all positions in window sequence. The P-value is computed with Fisher’s exact function from Python scipy package. Table S2: Complete SNP specificity ranking for all positions. Table S3: The feature distribution in HMpre feature space. Algorithm S1: SNP Specificity Identification Algorithm. Figure S1: Distribution of feature importance scores in XGBoost Classifier learning stage. (PDF 323 kb)

## Abbreviations

CPD: Chemical property with density feature
m^6^A: N^6^-Methylation
MRMR: Max-Relevance Min-Redundancy algorithm
NGS: Next generation sequence
PTM: Post-transcription modifications
SNP: Singe nucleotide polymorphism

## References

[1] Machnicka MA, Milanowska K, Osman Oglou O, Purta E, Kurkowska M, Olchowik A, Januszewski W, Kalinowski S, Dunin-Horkawicz S, Rother KM (2012). Modomics: a database of rna modification pathways—2013 update. Nucleic Acids Res.

[2] Motorin Y, Helm M (2011). Rna nucleotide methylation. Wiley Interdiscip Rev RNA.

[3] Wu R, Jiang D, Wang Y, Wang X (2016). N6-methyladenosine (m6a) methylation in mrna with a dynamic and reversible epigenetic modification. Mol Biotechnol.

[4] Fu Y, Dominissini D, Rechavi G, He C (2014). Gene expression regulation mediated through reversible m6a rna methylation. Nat Rev Genet.

[5] Wan Y, Tang K, Zhang D, Xie S, Zhu X, Wang Z, Lang Z (2015). Transcriptome-wide high-throughput deep m6a-seq reveals unique differential m6a methylation patterns between three organs in arabidopsis thaliana. Genome Biol.

[6] Chen W, Tran H, Liang Z, Lin H, Zhang L (2015). Identification and analysis of the n 6-methyladenosine in the saccharomyces cerevisiae transcriptome. Sci Rep.

[7] Deng X, Chen K, Luo G-Z, Weng X, Ji Q, Zhou T, He C (2015). Widespread occurrence of n6-methyladenosine in bacterial mrna. Nucleic Acids Res.

[8] Huang W, Xiong J, Yang Y, Liu S-M, Yuan B-F, Feng Y-Q (2015). Determination of dna adenine methylation in genomes of mammals and plants by liquid chromatography/mass spectrometry. RSC Adv.

[9] Dominissini D, Moshitch-Moshkovitz S, Schwartz S, Salmon-Divon M, Ungar L, Osenberg S, Cesarkas K, Jacob-Hirsch J, Amariglio N, Kupiec M (2012). Topology of the human and mouse m6a rna methylomes revealed by m6a-seq. Nature.

[10] Meyer KD, Saletore Y, Zumbo P, Elemento O, Mason CE, Jaffrey SR (2012). Comprehensive analysis of mrna methylation reveals enrichment in 3’utrs and near stop codons. Cell.

[11] Wang Y, Li Y, Toth JI, Petroski MD, Zhang Z, Zhao JC (2014). N6-methyladenosine modification destabilizes developmental regulators in embryonic stem cells. Nat Cell Biol.

[12] Liu J, Yue Y, Han D, Wang X, Fu Y, Zhang L, Jia G, Yu M, Lu Z, Deng X (2014). A mettl3-mettl14 complex mediates mammalian nuclear rna n6-adenosine methylation. Nat Chem Biol.

[13] Ping X-L, Sun B-F, Wang L, Xiao W, Yang X, Wang W-J, Adhikari S, Shi Y, Lv Y, Chen Y-S (2014). Mammalian wtap is a regulatory subunit of the rna n6-methyladenosine methyltransferase. Cell Res.

[14] Wang X, Zhao BS, Roundtree IA, Lu Z, Han D, Ma H, Weng X, Chen K, Shi H, He C (2015). N6-methyladenosine modulates messenger rna translation efficiency. Cell.

[15] Bokar JA (2005). The biosynthesis and functional roles of methylated nucleosides in eukaryotic mrna. Fine-tuning of RNA Functions by Modification and Editing.

[16] Shen F, Huang W, Huang J-T, Xiong J, Yang Y, Wu K, Jia G-F, Chen J, Feng Y-Q, Yuan B-F (2015). Decreased n6-methyladenosine in peripheral blood rna from diabetic patients is associated with fto expression rather than alkbh5. J Clin Endocrinol Metab.

[17] Yang Y, Huang W, Huang J-T, Shen F, Xiong J, Yuan E-F, Qin S-s, Zhang M, Feng Y-Q, Yuan B-F (2016). Increased n6-methyladenosine in human sperm rna as a risk factor for asthenozoospermia. Sci Rep.

[18] Choi J, Ieong K-W, Demirci H, Chen J, Petrov A, Prabhakar A, O’leary SE, Dominissini D, Rechavi G, Soltis SM (2016). N6-methyladenosine in mrna disrupts trna selection and translation-elongation dynamics. Nat Struct Mol Biol.

[19] Tsai K, Courtney DG, Cullen BR (2018). Addition of m6a to sv40 late mrnas enhances viral structural gene expression and replication. PLoS Pathog.

[20] Lichinchi G, Gao S, Saletore Y, Gonzalez GM, Bansal V, Wang Y, Mason CE, Rana TM (2016). Dynamics of the human and viral m6a rna methylomes during hiv-1 infection of t cells. Nat Microbiol.

[21] Riquelme Barrios SA, Pereira-Montecinos C, Valiente-Echeverría F, Soto-Rifo R (2018). Emerging roles of n6-methyladenosine on hiv-1 rna metabolism and viral replication. Front Microbiol.

[22] Lichinchi G, Zhao BS, Wu Y, Lu Z, Qin Y, He C, Rana TM (2016). Dynamics of human and viral rna methylation during zika virus infection. Cell Host Microbe.

[23] Zhang C, Samanta D, Lu H, Bullen JW, Zhang H, Chen I, He X, Semenza GL (2016). Hypoxia induces the breast cancer stem cell phenotype by hif-dependent and alkbh5-mediated m6a-demethylation of nanog mrna. Proc Natl Acad Sci.

[24] Keith G (1995). Mobilities of modified ribonucleotides on two-dimensional cellulose thin-layer chromatography. Biochimie.

[25] Zheng G, Dahl JA, Niu Y, Fedorcsak P, Huang C-M, Li CJ, Vgb CB, Shi Y, Wang W-L, Song S-H (2013). Alkbh5 is a mammalian rna demethylase that impacts rna metabolism and mouse fertility. Mol Cell.

[26] Liu H, Flores MA, Meng J, Zhang L, Zhao X, Rao MK, Chen Y, Huang Y (2014). Met-db: a database of transcriptome methylation in mammalian cells. Nucleic Acids Res.

[27] Chen W, Feng P, Ding H, Lin H, Chou K-C (2015). irna-methyl: identifying n6-methyladenosine sites using pseudo nucleotide composition. Anal Biochem.

[28] Chen W, Xing P, Zou Q (2017). Detecting n6-methyladenosine sites from rna transcriptomes using ensemble support vector machines. Sci Rep.

[29] Schwartz S, Agarwala SD, Mumbach MR, Jovanovic M, Mertins P, Shishkin A, Tabach Y, Mikkelsen TS, Satija R, Ruvkun G (2013). High-resolution mapping reveals a conserved, widespread, dynamic mrna methylation program in yeast meiosis. Cell.

[30] Zhou Y, Zeng P, Li Y-H, Zhang Z, Cui Q (2016). Sramp: prediction of mammalian n6-methyladenosine (m6a) sites based on sequence-derived features. Nucleic Acids Res.

[31] Chen W, Tang H, Lin H (2017). Methyrna: a web server for identification of n6-methyladenosine sites. J Biomol Struct Dyn.

[32] Xing P, Su R, Guo F, Wei L (2017). Identifying n6-methyladenosine sites using multi-interval nucleotide pair position specificity and support vector machine. Sci Rep.

[33] Ke S, Alemu EA, Mertens C, Gantman EC, Fak JJ, Mele A, Haripal B, Zucker-Scharff I, Moore MJ, Park CY (2015). A majority of m6a residues are in the last exons, allowing the potential for 3’utr regulation. Gene Dev.

[34] Linder B, Grozhik AV, Olarerin-George AO, Meydan C, Mason CE, Jaffrey SR (2015). Single-nucleotide-resolution mapping of m6a and m6am throughout the transcriptome. Nat Methods.

[35] Xiang S, Yan Z, Liu K, Zhang Y, Sun Z (2016). Athmethpre: a web server for the prediction and query of mrna m 6 a sites in arabidopsis thaliana. Mol BioSyst.

[36] Chen W, Feng P, Ding H, Lin H (2016). Identifying n6-methyladenosine sites in the arabidopsis thaliana transcriptome. Mol Gen Genomics.

[37] Wang X, Yan R (2018). Rfathm6a: a new tool for predicting m6a sites in arabidopsis thaliana. Plant Mol Biol.

[38] Liu Z, Xiao X, Yu D-J, Jia J, Qiu W-R, Chou K-C (2016). prnam-pc: Predicting n6-methyladenosine sites in rna sequences via physical–chemical properties. Anal Biochem.

[39] Zhang M, Sun J-W, Liu Z, Ren M-W, Shen H-B, Yu D-J (2016). Improving n 6-methyladenosine site prediction with heuristic selection of nucleotide physical–chemical properties. Anal Biochem.

[40] Li G-Q, Liu Z, Shen H-B, Yu D-J (2016). Targetm6a: Identifying n6-methyladenosine sites from rna sequences via position-specific nucleotide propensities and a support vector machine. IEEE Trans Nanobioscience.

[41] Wan S, Duan Y, Zou Q (2017). Hpslpred: An ensemble multi-label classifier for human protein subcellular location prediction with imbalanced source. Proteomics.

[42] Zou Q, Wan S, Ju Y, Tang J, Zeng X (2016). Pretata: predicting tata binding proteins with novel features and dimensionality reduction strategy. BMC Syst Biol.

[43] Peng H, Long F, Ding C (2005). Feature selection based on mutual information criteria of max-dependency, max-relevance, and min-redundancy. IEEE Trans Pattern Anal Mach Intell.

[44] Zheng Y, Nie P, Peng D, He Z, Liu M, Xie Y, Miao Y, Zuo Z, Ren J (2017). m6avar: a database of functional variants involved in m6a modification. Nucleic Acids Res.

[45] Sauna ZE, Kimchi-Sarfaty C (2011). Understanding the contribution of synonymous mutations to human disease. Nat Rev Genet.

[46] Zou Q, Zeng J, Cao L, Ji R (2016). A novel features ranking metric with application to scalable visual and bioinformatics data classification. Neurocomputing.

[47] Saeys Y, Inza I, Larrañaga P (2007). A review of feature selection techniques in bioinformatics. Bioinformatics.

[48] Chen W, Tang H, Ye J, Lin H, Chou K-C (2016). irna-pseu: Identifying rna pseudouridine sites. Mol Ther—Nucleic Acids.

[49] Yu D-J, Hu J, Huang Y, Shen H-B, Qi Y, Tang Z-M, Yang J-Y (2013). Targetatpsite: a template-free method for atp-binding sites prediction with residue evolution image sparse representation and classifier ensemble. J Comput Chem.

[50] Hu J, He X, Yu D-J, Yang X-B, Yang J-Y, Shen H-B (2014). A new supervised over-sampling algorithm with application to protein-nucleotide binding residue prediction. PLoS ONE.

[51] Song L, Li D, Zeng X, Wu Y, Guo L, Zou Q (2014). ndna-prot: identification of dna-binding proteins based on unbalanced classification. BMC Bioinformatics.

[52] Wei Z-S, Han K, Yang J-Y, Shen H-B, Yu D-J (2016). Protein–protein interaction sites prediction by ensembling svm and sample-weighted random forests. Neurocomputing.

[53] Liu G-H, Shen H-B, Yu D-J (2016). Prediction of protein–protein interaction sites with machine-learning-based data-cleaning and post-filtering procedures. J Membr Biol.

[54] Lin C, Chen W, Qiu C, Wu Y, Krishnan S, Zou Q (2014). Libd3c: ensemble classifiers with a clustering and dynamic selection strategy. Neurocomputing.

[55] He H, Garcia EA (2009). Learning from imbalanced data. IEEE Trans Knowl Data Eng.

[56] Chen T, Guestrin C. Xgboost: A scalable tree boosting system. In: Proceedings of the 22nd ACM SIGKDD International Conference on Knowledge Discovery and Data Mining. ACM: 2016. p. 785–794.

[57] Sun W-J, Li J-H, Liu S, Wu J, Zhou H, Qu L-H, Yang J-H (2015). Rmbase: a resource for decoding the landscape of rna modifications from high-throughput sequencing data. Nucleic Acids Res.

