# Supplementary material for: Imbalance learning for the prediction of N6-Methylation sites in mRNAs
Source: BMC Genomics. 2018 Aug 1;19:574. doi: 10.1186/s12864-018-4928-y (PMC6090857; doi:10.1186/s12864-018-4928-y)
Supplement: Supplementary file 2 — Supplementary Tables, Algorithm and Figure. Table S1: The result of Fisher’s exact test on training data. The SNP variant states of positive and negative samples are counted respectively at all positions in window sequence. The P-value is computed with Fisher’s exact function from Python scipy package. Table S2: Complete SNP specificity ranking for all positions. Table S3: The feature distribution in HMpre feature space. Algorithm S1: SNP Specificity Identification Algorithm. Figure S1: Distribution of feature importance scores in XGBoost Classifier learning stage. (PDF 323 kb) [file 12864_2018_4928_MOESM2_ESM.pdf]

Supplementary materials

Table S1. Fisher's Exact Test Result on Training Data

Position	Positive		Negative		P-value	log(P-value)
	0	1	0	1		
**-25**	22938	3574	237968	33246	1.1392E-08	7.943411816
**-24**	23046	3466	238766	32448	1.6362E-07	6.786161828
**-23**	22873	3639	236820	34394	1.4368E-06	5.842594305
**-22**	23127	3385	237734	33480	4.6296E-02	1.334459712
**-21**	23080	3432	238822	32392	2.1623E-06	5.665074778
**-20**	22956	3556	237311	33903	2.2359E-05	4.650551911
**-19**	23028	3484	238524	32690	3.0275E-07	6.51891752
**-18**	23179	3333	239392	31822	6.1149E-05	4.213613994
**-17**	23008	3504	236945	34269	6.9935E-03	2.155306589
**-16**	23071	3441	238012	33202	5.2626E-04	3.278799655
**-15**	23109	3403	238447	32767	3.6345E-04	3.439555375
**-14**	22890	3622	236473	34741	8.5695E-05	4.067044889
**-13**	23017	3495	237358	33856	1.0991E-03	2.958949538
**-12**	23112	3400	238708	32506	7.1291E-05	4.146964601
**-11**	22977	3535	237141	34073	3.3893E-04	3.469884854
**-10**	23116	3396	238467	32747	5.0566E-04	3.296143673
**-9**	23203	3309	239626	31588	6.3040E-05	4.200380539
**-8**	23035	3477	237721	33493	3.4257E-04	3.465256475
**-7**	23160	3352	238377	32837	1.1075E-02	1.95565113
**-6**	23203	3309	239175	32039	1.4083E-03	2.85131802
**-5**	22963	3549	236486	34728	7.1140E-03	2.147883425
**-4**	22918	3594	237319	33895	9.2043E-07	6.036007285
**-3**	23134	3378	239047	32167	2.8166E-05	4.55028225
**-2**	23520	2992	245937	25277	2.3900E-24	23.62160012

**-1**	24019	2493	249450	21764	1.5465E-14	13.81065704
**m^{6}A**	24282	2230	250170	21044	1.8411E-04	3.734925499
**1**	22970	3542	234720	36494	6.7129E-01	0.173092105
**2**	23288	3224	234679	36535	1.4922E-09	8.826160222
**3**	22898	3614	234283	36931	9.4765E-01	0.02335424
**4**	22851	3661	235042	36172	3.1919E-02	1.495950385
**5**	23055	3457	236480	34734	2.8121E-01	0.550972658
**6**	23086	3426	237858	33356	3.3538E-03	2.47446888
**7**	23025	3487	236516	34698	9.5986E-02	1.017791585
**8**	23134	3378	238543	32671	9.8694E-04	3.005710269
**9**	23202	3310	239179	32035	1.3133E-03	2.881649017
**10**	23050	3462	237082	34132	2.7208E-02	1.56530172
**11**	23058	3454	237720	33494	1.4655E-03	2.834011725
**12**	23140	3372	239222	31992	1.1026E-05	4.957564887
**13**	23030	3482	237314	33900	3.0605E-03	2.514212071
**14**	23183	3329	238305	32909	4.5754E-02	1.339568637
**15**	23092	3420	239299	31915	7.3293E-08	7.134938054
**16**	22993	3519	237004	34210	2.1694E-03	2.663658376
**17**	23130	3382	237953	33261	2.0281E-02	1.692903976
**18**	23200	3312	238704	32510	1.6251E-02	1.789117825
**19**	22976	3536	236972	34242	9.4642E-04	3.023916026
**20**	23227	3285	237804	33410	7.3193E-01	0.13552882
**21**	23225	3287	239274	31940	2.9910E-03	2.524182748
**22**	23077	3435	237502	33712	1.3765E-02	1.861217582
**23**	23205	3307	238094	33120	2.1604E-01	0.665466371
**24**	23223	3289	239057	32157	8.7203E-03	2.059467838
**25**	23024	3488	237090	34124	7.5174E-03	2.123929588

(This table shows the Fisher's exact test results on training data. There are 26512 positive and 271214 negative samples in training dataset, and we count the SNP variant states (0 or 1) of each position in positive and negative samples respectively. Then Fisher's exact test is used to evaluate the distribution

difference of SNP states between positive and negative sample, then a P-value is calculated to demonstrate the difference level. A lower P-value means a higher SNP state distribution difference at the position.)

Table S2. Position SNP Specificity Ranking with MRMR and Fisher's Test

position	Fisher's exact Test	MRMR	Average	Rank
**-2**	1	1	1	1
**-1**	2	5	3.5	2
**-24**	6	7	6.5	3
**-21**	10	4	7	4
**-19**	7	12	9.5	5
**2**	3	23	13	6
**-25**	4	24	14	7
**-11**	19	9	14	8
**-4**	8	21	14.5	9
**-15**	21	11	16	10
**-9**	15	17	16	11
**-23**	9	25	17	12
**16**	30	8	19	13
**25**	36	2	19	14
**-13**	26	15	20.5	15
**m^6A**	18	27	22.5	16
**11**	29	16	22.5	17
**18**	40	6	23	18
**-18**	14	33	23.5	19
**6**	33	14	23.5	20
**15**	5	42	23.5	21
**-20**	12	36	24	22
**-6**	28	20	24	23
**9**	27	22	24.5	24
**-3**	13	37	25	25
**13**	32	18	25	26
**-12**	16	35	25.5	27
**-8**	20	31	25.5	28
**1**	49	3	26	29
**22**	39	13	26	30
**-17**	34	19	26.5	31
**19**	24	32	28	32
**-14**	17	40	28.5	33
**12**	11	48	29.5	34
**20**	50	10	30	35
**-16**	23	41	32	36
**-10**	22	43	32.5	37
**24**	37	28	32.5	38
**21**	31	38	34.5	39
**14**	44	30	37	40
**7**	46	29	37.5	41

**8**	25	51	38	42
**3**	51	26	38.5	43
**10**	42	-2	20	44
**5**	48	34	41	45
**-5**	35	49	42	46
**-7**	38	47	42.5	47
**17**	41	45	43	48
**-22**	45	44	44.5	49
**4**	43	50	46.5	50
**23**	47	46	46.5	51

Table S3. Features Space used in HMpre

**Feature No.**	**Feature name**
1-204	4-bit Binary Encoding
205-408	Chemical Property with Density function
409-488	K-mer (2-mer, 3- mer)
489-500	SNP
501-502	Site Location
503-509	Information Theory Feature

Table S4. 10-fold cross validation results of conventional feature combinations with new features

**Feature Space**	**precision**	**recall**	***F1***	***ACC***	***MCC***
4-bit +3 new	0.2577	0.5217	0.345	0.8236	0.2777
OPF +3 new	0.2601	0.5083	0.3441	0.8275	0.2757
K-mer + 3 new	0.2098	0.3996	0.2751	0.8124	0.1916
4-bit +OPF +3 new	0.2598	0.2586	0.3445	0.8268	0.2763
4-bit +K-mer +3 new	0.2651	0.5311	0.3536	0.8271	0.288
OPF +K-mer +3 new	0.2664	0.5187	0.352	0.8299	0.2852
Proposed feature space	0.2669	0.5248	0.3538	0.8293	0.2877

(This table shows 10-fold cross validation results of different conventional feature combinations with new features. As is shown in the table, a single conventional feature with 3 new features has relatively lower performance than combinational conventional features. However, the proposed feature space achieves the best overall performance with highest precision, recall, F1 and ACC, so the three conventional features are all important in the prediction.)

Algorithm S1. SNP Specificity Identification Algorithm

Algorithm: SNP Specificity Identification Algorithm

Input: 51-bit SNP variant sequences of *n* training samples and *t* test samples, each sequence is (v_1, v_2, …, v_{51}) where v_k denote the SNP state (0 or 1, variant or not) of *k*-th position in samples; training sample label set *C*.

Combine the SNP sequences of training samples into *n51 Matrix S_0;**
for *i* = 1 to 51 do
Calculate probabilistic density function $p(x_i)$ for column *i* in S_0;
Calculate $p(y)$ for *C* and joint probability $p\ (x_i, y)$;
Define mutual information: $M_i(x_i; y) = \sum_{x_i=0}^{1} \sum_{y=0}^{1} p\ (xi, y) log \frac{p\ (xi,y)}{p\ (xi) p\ (y)}$
end for
Give the feature number *m* of target subsets *S*;
for *j* = 1 to *m* do
for s_i in S_0 do
Put s_i into *S*
Dependency between *S* and *C*: $D = \frac{1}{|S|} \sum_{x_i \in S} M_i(x_i; y)$
Redundancy among *S*: $R = \frac{1}{|S|^2} \sum_{x_i, x_j \in S} M_i(x_i; x_j)$
Computing $\emptyset = D - R$
end for
take the s_m which has maximum *Ø* as the *j*-th column of *S*
mrmr rank(s_m)=*j*
importance(s_m)= ∅
Remove s_m from S_0
end for
for *k* =1 to 51 do
count number of variant at position *k* in datasets:
training samples have *p* variants and *q* non-variants
testing samples have *p'* variants and *q'* non-variants
p-value = fisher test (*p, q, p', q'*)
end for
fisher test rank = sort(p-value)
specificity rank of position *v* = average (mrmr rank(*v*), fisher test rank(*v*))
take top *12* SNP-specific positions as *SNP feature*

Output: *SNP feature*

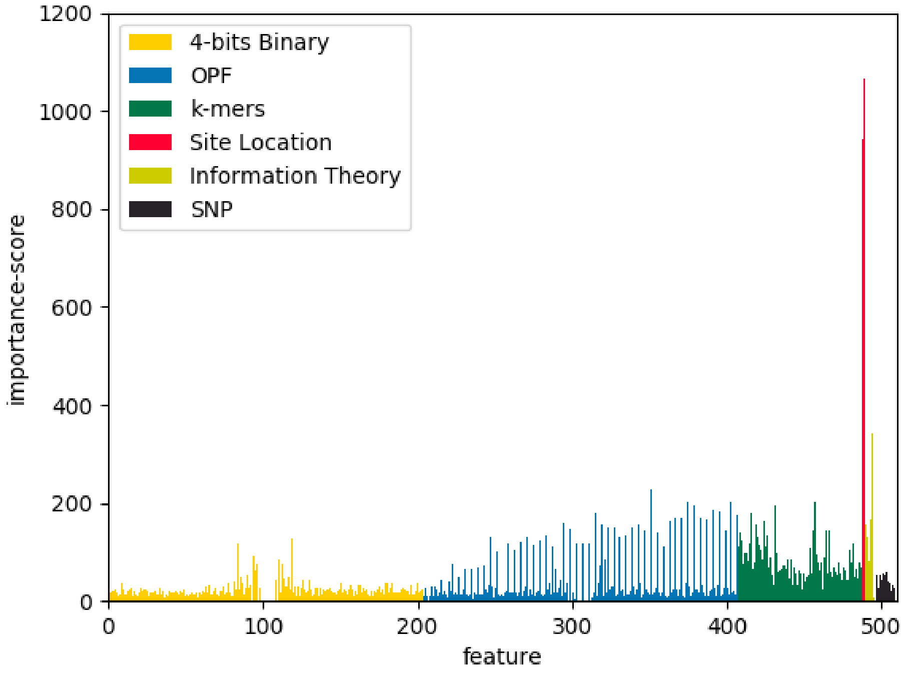

Fig. S1. Distribution of feature importance scores in XGBoost Classifier learning stage. here are 508 features in total and several colours represent features constructed by different coding algorithms. The importance scores of most features are less than 50 in 4-bit Binary and OPF features. Even some features have an average score much less than average, and these features correspond to the m6A and adjacent two sites. The three centre positions of samples are always ’AAC’ or ’GAC’, so their importance scores are indeed low. And the highest three features are frequency of adenine in transcript, relative site location and relative entropy features. Meanwhile, density features in OPF has are latively high importance, which indicates the nucelotide density of each site make sense in prediction. The importance scores of K-mer and SNP features are almost more than 4-bit Binary and Chemical Property features, which have low significance in the model tree construction.
